# A Fault Diagnostic Scheme Based on Capsule Network for Rolling Bearing under Different Rotational Speeds

**DOI:** 10.3390/s20071841

**Published:** 2020-03-26

**Authors:** Linjie Li, Mian Zhang, Kesheng Wang

**Affiliations:** 1 School of Mechanical and Electrical Engineering, University of Electronic Science and Technology of China, Chengdu 611731, China; linjieli_uestc@163.com; 2 Tianjin Key Laboratory of the Design and Intelligent Control of the Advanced Mechanical System, Tianjin University of Technology, Tianjin 300384, China; zoommian@foxmail.com; 3 National Demonstration Center for Experimental Mechanical and Electrical Engineering Education, Binshuixidao 391, Xiqing District, Tianjin 300384, China

**Keywords:** fault diagnosis, capsule network, two-direction signals, end-to-end scheme, rolling bearing, different rotational speeds

## Abstract

Deep learning-based intelligent fault diagnosis methods have attracted increasing attention for their automatic feature extraction ability. However, existing works are usually under the assumption that the training and test dataset share similar distributions, which unfortunately always violates real practice due to the variety of working conditions. In this paper, an end-to-end scheme of joint use of two-direction signals and capsule network (CN) is proposed for fault diagnosis of rolling bearing. With the help of the superior ability of CN in capturing the spatial position information between features, more valuable information can be mined. Aiming to eliminate the influence of different rotational speeds, vertical and horizontal vibration signals are fused as the input to CN, so that invariant features can be extracted automatically from the raw signals. The effectiveness of the proposed method is verified by experimental data of rolling bearing under different rotational speeds and compared with a deep convolutional neural network (DCNN). The results demonstrate that the proposed scheme is able to recognize the fault types of rolling bearing under scenarios of different rotational speeds.

## 1. Introduction

Rolling bearing is one of the key components of rotating machines. However, due to the hostile working conditions, the rolling bearing is prone to failure and may cause the breakdown of the machines, or even catastrophic consequences. Therefore, effective fault diagnosis plays an indispensable role to ensure the safety of machinery and reduce unnecessary cost. With the rapid development of fault diagnostic technologies, a large number of effective methods have been proposed in the past few years, including temperature, oil-debris analysis, vibration and acoustic emission, etc. [1]. Among them, vibration analysis is a widely used method for rotating machinery due to its exceptional adaptability and easy-to-get signal. Many methods have been proposed based on signal processing techniques, data-driven techniques and the combination of both [2,3,4,5].

Traditional intelligent methods for fault diagnosis, such as support vector machine (SVM) [6], back propagation neural network (BPNN) [7], and random forest [8], require manual intervention and expert knowledge, which significantly depend on the quality of manual feature extraction and selection [9]. With the evolution of machine learning techniques, deep learning has shown considerable power in the field of fault diagnosis [10], which may overcome the inherent limitations of traditional intelligent methods. Deep learning method can largely eliminate the procedure of manual feature extraction and avoid the dependence on the prior knowledge about signal processing techniques, which is an end-to-end method [11]. For example, Lei et al. [12] used a deep neural network (DNN) trained by frequency spectra for fault diagnosis. The proposed method was tested by using different datasets from rolling element bearings and planetary gearboxes and compared with back propagation neural network (BPNN)-based method. The results showed that the DNN-based method has superior diagnosis accuracy and adaptability. Zhang et al. [13] proposed a deep convolutional neural network with a wide first-layer kernel. The input of the network is the one-dimension (1D) raw vibration signal without any pre-signal processing. The results showed that the proposed network architecture featured high accuracy. Moreover, many convolutional neural network methods based on two-dimension (2D) image representation have been developed, such as in [14,15]. In these methods, the raw vibration signal is first transformed into the 2D time-frequency images by the signal processing technique, such as short time Fourier transform (STFT). Then the 2D images are inputted to train the network for classification purpose. Although this approach shows high accuracy, yet the computational time to train the model by 2D images is still quite long and therefore, to some extent, not very practical.

The above deep learning approaches have shown their superior performance compared with traditional intelligent fault diagnosis methods. However, these fault diagnosis methods are basically proposed under an assumption that the training and testing vibration signals are collected with the same statistical data distribution, unfortunately it is almost impossible in real-world application. Specifically, for rotational machines, one of the key reasons to lead to the differences between statistical data distribution is due to the time-varying non-stationery rotational speeds. Focused on this problem, studies have contributed to eliminating the influences of speed manually [16], including order tracking, holographic spectrum, speed normalization and domain adaptation, etc. [17,18,19,20]. For order tracking, holographic spectrum and speed normalization techniques, the information of speed is required to reduce influences.

Domain adaptation, however, is a transfer learning method, which can align the distributions between source and target domain data without using speed information before training the model. It makes even less data information input to the model while remaining high classification accuracy. For example, Long et al. [20] used maximum mean discrepancy (MMD) to joint distribution alignments in deep networks. Qian et al. [21] proposed a novel transfer learning method to align both the marginal and conditional distributions of both source and target domain datasets. In addition, the method is proved to be effective for fault diagnosis on roller bearing and gearbox. Transfer learning-based domain adaptation is indeed an effective approach to settle the problem of variable working conditions. However, the target and source domain need to align the distribution in advance and the data of target domain must participate in the training process of the model. In this paper, we aim at eliminating the influences of rotational speed between measurements automatically. The unique ability of capsule network (CN) in capturing the spatial variation of the data is explored [22]. A capsule network-based approach, in which two-directional signals are collected for training, is proposed. To make full use of the two-direction signals, data fusion methods, including no processing, channel overlay and stack banding, are used. Channel overlay and stack banding can fuse the two-direction signals to form a two-dimensional data, which will be introduced in detail in the later section. The experimental results show that the network can extract the invariable features from the raw vibration signal, and show a better performance than traditional CNN method under different rotational speeds.

The remainder of this paper is organized as follows. The architecture and principle of the capsule network are introduced in Section 2. Section 3 gives an overview of the proposed scheme. In Section 4, the application of the proposed scheme on rolling bearing is presented in detail. Then the performance is discussed. Section 5 concludes the paper.

## 2. Capsule Neural Network

Traditional convolutional neural network (CNN), called LeNet, was first proposed by Lecun in 1998 and showed excellent performance on image recognition [23]. Then, some improved CNNs with deeper layers were consequently designed and achieved satisfactory results on image recognition, including AlexNet, VGGNet, GoogLeNet, ResNet [24,25]. Although these networks may obtain high accuracy for the classification of images, the spatial information in image cannot be perfectly captured, in other words, the changes of positions or angles of objects in an image can lead to an unsatisfactory classification result. To this end, capsule network with a so-called dynamic routing is proposed to solve this very problem in CNN.

A general CNN is mainly composed of convolutional layers, pooling layers, fully connected layer and activation function. The feature of input is first extracted by convolutional layers and pooling layers. Then, a fully connected layer is applied for classification [26]. The application of convolution and pooling operation is the key steps to contribute to a deeper layer of CNN, which reduces the number of trainable parameters by parameter sharing. The pooling layer can reduce the size of data because the features of the image can stay unchanged after pooling. However, Hinton [27] indicates that the usage of max-pooling may also lead to a deficiency that the location relationship of features may be ignored. Focused on this deficiency, Hinton and his assistant proposed a novel neural network in 2017, i.e., capsule network (CN). This network integrates the advantages of CNN in non-linear feature extraction and retains the positional information by using the module length of vector to describe the probability of the predicted result [27].

### 2.1. The Architecture of Capsule Network

**Figure 1:**
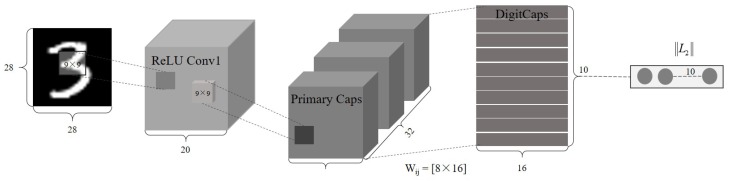
Architecture of a capsule network with 3 layers.

**Figure 2:**
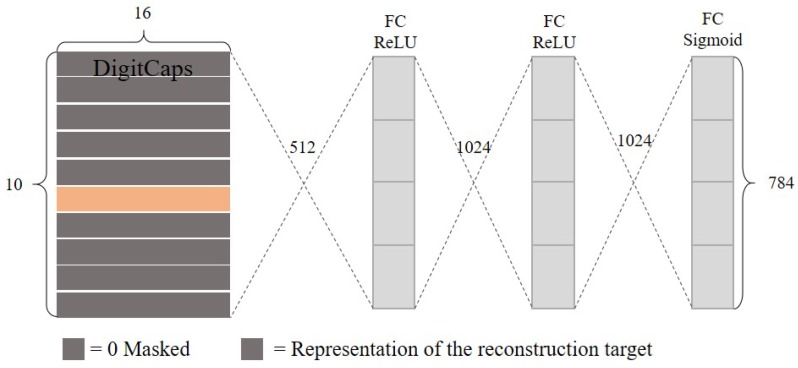
Decoder structure to reconstruct the inputs.

A simple CN architecture for image recognition is shown in Figure 1 and Figure 2. It includes four parts, convolution, primary capsule, digit capsule and decoder. Each part is briefly introduced in this section. As an example, the input size is set as 28 × 28 and the number for classification is 10 [27].

(1) Convolution layer

The first layer of CN is a traditional convolution layer with “ReLU” activation function, which is applied to extract the preliminary feature. After the initial convolutional operation with kernel size of 9 × 9 and number of 256, the extracted features with size of 20 × 20 × 256 are then used as inputs to the primary capsules. In addition, pooling operation is not appended after convolution to avoid the information loss.   

(2) Primary capsule layer

In this layer, the capsule is preliminarily developed. Another convolution operation with the kernel size of 9 × 9 and number of 256 is first applied. The output after the convolution is then reshaped into capsules with size of 6 × 6 × 32 × 8, which means the dimensionality of each capsule is 8. Squashing function, which will be interpreted later, is used to squash the vector length within the range of 0–1. This layer can obtain low-level features in a vector form.

(3) Digit capsule layer

This layer is to get high-level features in vector form and is fully connected with primary capsule layer between vectors by a matrix $W_{ij}$. Each capsule from previous layer is multiplied by $W_{ij}$ with a size of 8 × 16, which will be interpreted in detail later. Therefore, the output size of this layer is 6 × 6 × 32 × 16. In addition, a dynamic routing algorithm, which also will be introduced later, is applied between primary and digit capsules. The weight parameter of each capsule can be determined by dynamic routing instead of backpropagation. After dynamic routing several times, the final capsules with a size of 10 × 16 are then sent to two different branches: one to compute the length for classification and the other to reconstruct the input images.   

(4) Decoder

Decoder is an extra layer in CN compared with CNN. It serves as the function very similar to an autoencoder. The capsule with maxlength from digit capsules is selected to input the 3 fully connected layers for reconstruction as described in Figure 2. The reconstruction loss is also considered to update the parameters. Therefore, the total loss of CN contains two parts, namely classification and reconstruction, and is the weighted sum of both.

### 2.2. The Principle of Capsule Network

In CN, many capsules with special significances, including orientation and length, are the key components of the network. The activation of the neuron within the capsule represents a variety of properties present in the image. These properties can include many different types of instantiation parameters in the image, such as posture, deformation, velocity, tone, and texture. A very special property of the capsule is the existence of an instance of a category in the image. Due to these unique capabilities of CN, in this work, we will explore this method to solve the problem of fault classification problem under different rotational speeds.

Compared with the traditional neural network, the output of neuron is a vector rather than a scalar, whose length can represent the estimated probability of the existence of the object. In addition, the non-linear activation function is replaced with a “squashing” function to ensure that short vectors are confined to almost zero length and long vectors are confined to a length slightly below one. Therefore, the length after squashing can be represented as the probability of a given character.

(1) $$v_{j}=\frac{{∥s_{j}∥}^{2}}{1+∥s_{j}{∥}^{2}}\frac{s_{j}}{∥s_{j}∥}$$

where $s_{j}$ is the total input of capsule j and $v_{j}$ is the its vector output.

**Figure 3:**
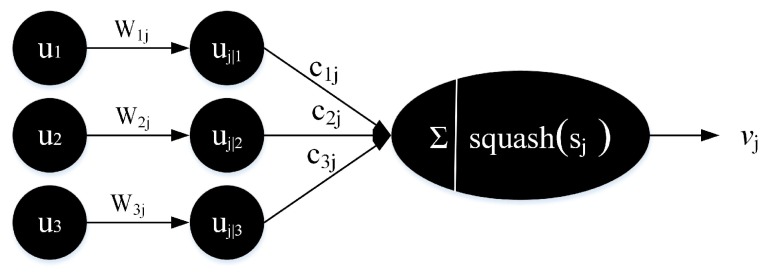
The computation of vector neuron.

The computational procedure of vector neuron $v_{j}$ of capsule j between primary capsule layer and digit capsule layer can be summarized as three steps and described in Figure 3. First, the initial vector $u_{i}$ from primary capsules is multiplied by weight matrix $W_{ij}$ to get the predicted vector $u_{j|i}$. Secondly, a weighted sum over all predicted vectors $u_{j|i}$ is performed to obtain the following vector $s_{j}$. Finally, the vector $s_{j}$ is transformed into vector $v_{j}$ using the "squashing" function.

(2) $$s_{j}=\Sigma c_{ij}u_{j|i},\;\;\;\;u_{j|i}=W_{ij}u_{i}$$

where $c_{ij}$ is called coupling coefficients and are determined by the iterative dynamic routing algorithm.

The coupling coefficients between capsule *i* and all the capsules in the layer above sum to 1 and are calculated through a “routing SoftMax” whose initial logits $b_{ij}$ are the log prior probabilities that capsule *i* should be coupled to capsule *j* [27].

(3) $$c_{ij}=\frac{exp(b_{ij})}{\Sigma exp(b_{ik})}$$

The key function of CN is to obtain the coupling coefficient $c_{ij}$ and it is determined by the iterative dynamic routing process. The detail of routing algorithm is shown in Algorithm 1, where *r* is the times of routing.
**Algorithm 1** Dynamic routing algorithm1:Procedure Routing ($u_{j|i},r,l$)2:for all capsule *i* in layer *l* and capsule *j* in layer (l+1):$b_{ij}\leftarrow 0$.3:for *r* iterations do4:    for all capsule *i* in layer *l*:$c_{i}\leftarrow$$SoftMax(b_{i})$5:    for all capsule *j* in layer (l+1):$s_{j}\leftarrow \Sigma_{i}c_{ij}u_{ij}$6:    for all capsule *j* in layer (l+1):$v_{j}\leftarrow$$squash(s_{j})$7:    for all capsule *i* in layer *l* and capsule *j* in layer $\left(l+1\right):b_{ij}\leftarrow b_{ij}+u_{j|i}\cdot v_{j}$8:return $v_{j}$

## 3. The Procedure of the Proposed Scheme

In this paper, an end-to-end scheme based on CN and two-dimensional (2D) input data is proposed for health state identification of the rolling bearing under different rotational speeds. Specifically, the 2D data is constructed by fusing the signals collected from two sensors perpendicular to each other. The detail of data fusion method will be introduced later. Taking the structured 2D data as input, 2D convolution is used in CN.

**Figure 4:**
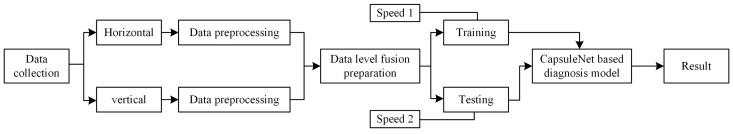
Flowchart of the proposed scheme.

Figure 4 displays the flowchart of the proposed method. In addition, there are four main steps:

(1) The vibration signals are collected by multiple sensors;
(2) Vibration signals from two-direction sensors, including horizontal and vertical, are normalized and sliced into segments;
(3) The segments from two directions are fused and then input to the CN;
(4) The CN is trained and tested using the data from different speeds. The test accuracy of the output result is used to evaluate the effectiveness of the proposed scheme.

## 4. Case Study and Experiment Results

**Figure 5:**
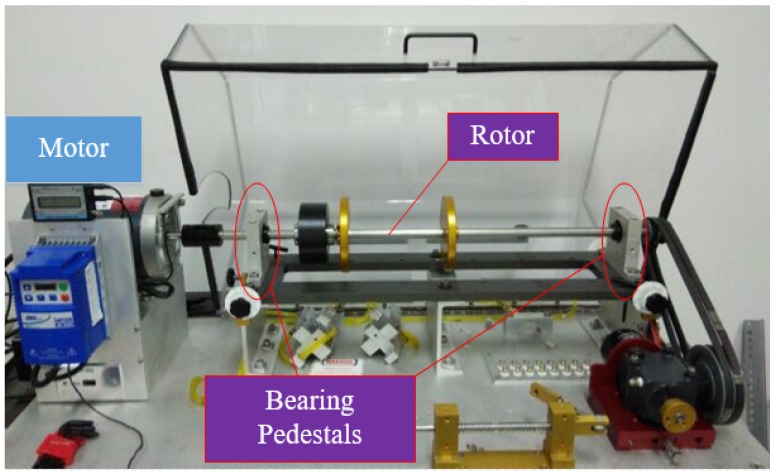
Comprehensive experimental platform for mechanical fault simulation.

In this section, the performance of the proposed method is tested. All data are collected from an MFS-MG test rig in University of Electronic Science and Technology of China (UESTC), as shown in Figure 5. The model of the capsule network is constructed in Python 3.6 using Pytorch and carried out on a PC with an Intel i7-8750H CPU, a GTX 1050 Ti GPU and operating system Win10.

### 4.1. Experimental Rolling Bearing Data Description

**Figure 6:**
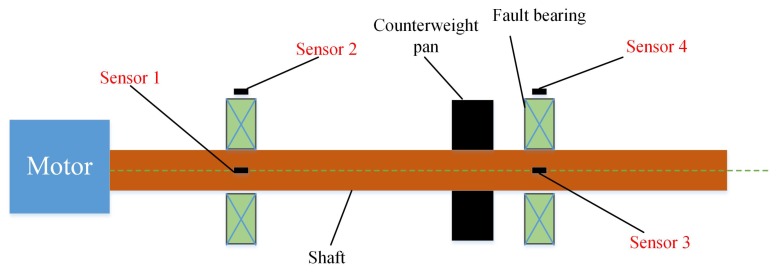
Schematic diagram of sensors layout.

To test the performance of the proposed scheme, we conduct experiments on the setup. As shown in Figure 5, the test rig mainly consists of a motor, two support bearing and a rotor. Experiments are conducted on the rolling bearing away from motor under four health states, including normal, inner race fault, rolling element fault and outer race fault. For each health state, vibration signals are collected when the drive motor operates under different rotational speeds to form up different domain datasets. The settings of speed include 800 rpm, 1500 rpm and 1800 rpm, resulting in different speed gaps. Therefore, the experimental dataset contains 4 different health states and 3 sets for each health state. In addition, four accelerometers are employed in this experiment. For each rolling bearing, two accelerometers are installed perpendicular to each other to collect vibration data in the horizontal and the vertical synchronously. To display the location of four sensors clearly, the layout is described using a schematic diagram, shown in Figure 6. As sensors 3 and 4 are directly installed on faulty bearing orthogonally, only vibration signals collected from these two sensors are used for follow-up work. The sampling frequency and measurement time are 20 KHz and around 3.2 s respectively, contributing to 65,536 sample points for each collected signal.

### 4.2. Data Processing

The collected signals need to be processed to input the network. First, the collected signals perform global normalization to speed up training. Then the preprocessing data are sliced into segments. Considering that a longer segment could be contain more information but fewer samples will be obtained, which may lead to overfitting issue during the network training, therefore, the size of each segment is set as 2048 to balance the length and the number of samples. In addition, the same vibration signal is sliced with overlap and no overlap respectively. In addition, the shift is set as 300 for overlap. Therefore, vibration signal with 65,536 points can provide 212 segments with overlap and 32 segments with no overlap. The obtained segments are then prepared for postprocessing.

**Figure 7:**
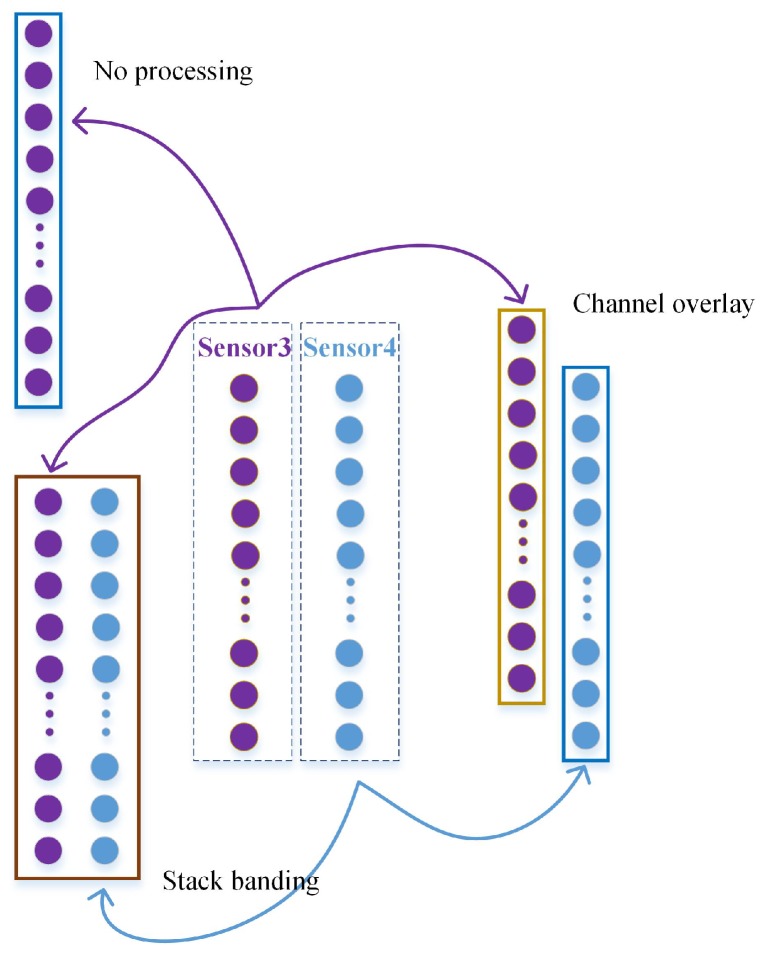
Three different methods of data fusion.

Three different data fusion methods are conducted on the segments from different sensors, as shown in Figure 7, namely no processing, channel overlay [28] and stack banding. No processing indicates that the segments from one single sensor are used and sensor 3 is selected in this study since training and test datasets are from the same rotational speed scenario and measured vibrations from either sensor 3 or 4 can be largely treated as the same input. The format of no processing is (1,2048) and one-dimensional convolution is used in the following studies. Channel overlay and stack banding fuse the two segments, namely the horizontal and the vertical vibration signals, to be an input data with the same period but different arrangement. The segments are first expanded into a three-dimensional representation, i.e., (1,1,2048), to in line with the input format of two-dimensional convolution (in_channel, in_width, in_height). Channel overlay refers to concatenating the segments of sensors 3 and 4 according to the “in_channel” to form up one data sample. In this way, the shape of the fused data sample will be (2,1,2048). Similar to channel overlay, stack banding concatenates the segments of sensors 3 and 4 according to the “in_width”, and results in a data of (1,2,2048). The whole dataset in detail is shown in Table 1. It should be noted that no processing, channel overlay, and stack bonding are represented by A, B and C, while three different speeds are represented by a, b and c.

### 4.3. Parameters of the Proposed Model

The model parameters will have a significant influence on the experimental results. Therefore, reasonable selection of model parameters needs to be studied here, including batch size, iteration, the size and number of convolutional kernels. To perform the process of parameter selection, dataset with the “stack banding” (C) is used. Considering the large speed gap scenario between set a and set c in Table 1, the training set is from set a with overlap while the test set is from set c without overlap. 

(1) Selection of batch size 

**Figure 8:**
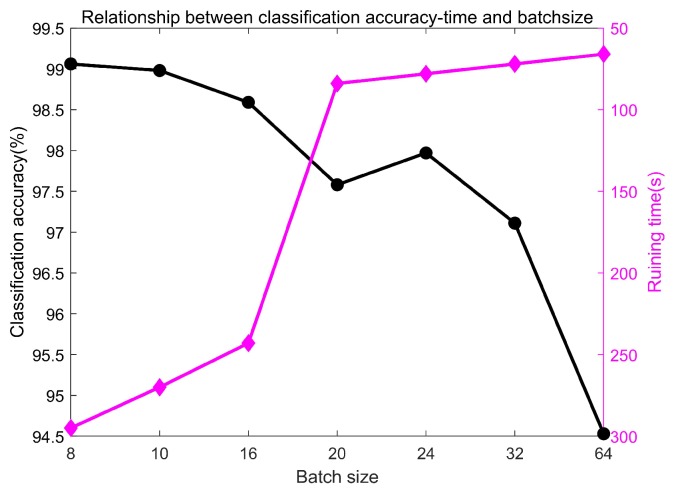
Relationship between classification accuracy-time and batch size.

Batch size refers to a certain number of samples at each training step. Reasonable selection of batch size can reduce training time and improve classification accuracy. Considering the limited memory capacity of computer and the number of training samples, the selected batch size are 8, 10, 16, 20, 24, 32 and 64, respectively. In addition, the number of iterations is preliminarily set as 70. For the convolution layer, two convolution kernels are used. As illustrated in [21], the high frequency noise commonly in vibration signals disturbs the local feature extraction, and the kernels of the first layer should be wide for suppressing the noise. Therefore, a wide kernel is used in the first layer and the size is 2 × 64, following a convolution kernel of 1 × 8 in the second layer. The experiment is repeated for ten times, and the average value is taken as the final classification accuracy. The relationship between the classification accuracy, time and batch size is shown in Figure 8. Balancing the training time and accuracy, the batch size is selected as 24.

(2) Selection of iteration times 

**Figure 9:**
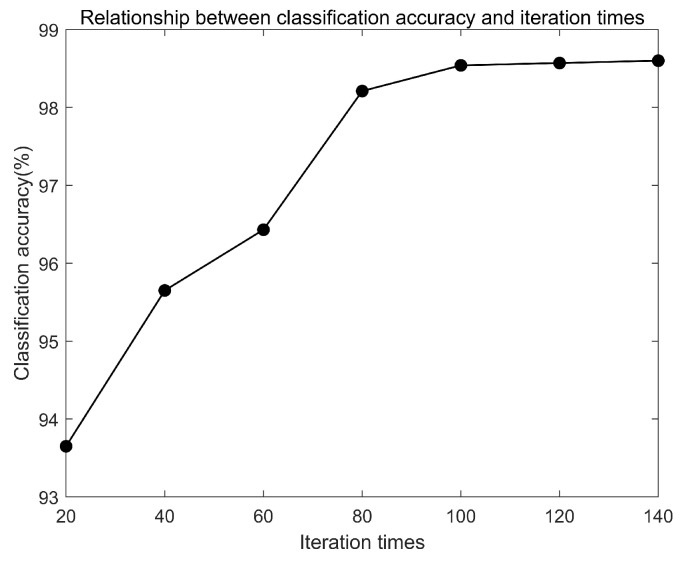
Relationship between classification accuracy and iteration times.

One iteration refers to forward propagation and back propagation once of the network. If the number of iterations is too small, the model is not well trained. When the number of iterations reaches a threshold, the model tends to be stable, which means that the accuracy will not increase, but time cost will increase with the increase of the number of iterations. Therefore, a suitable number of iterations needs to be chosen to reduce the training time. To simplify the complexity, the batch size is set as 24 according to the above and the convolution kernel is same. The relationship between classification accuracy and iteration times is shown in Figure 9. In Figure 9, the classification accuracy increases gradually with the increase of iteration times. When the iteration times exceed 100, the classification accuracy tends to be stable. To have a redundancy for the following parameter selection, the number of iterations is chosen as 120.

(3) Selection of the size and number of convolution kernels 

**Figure 10:**
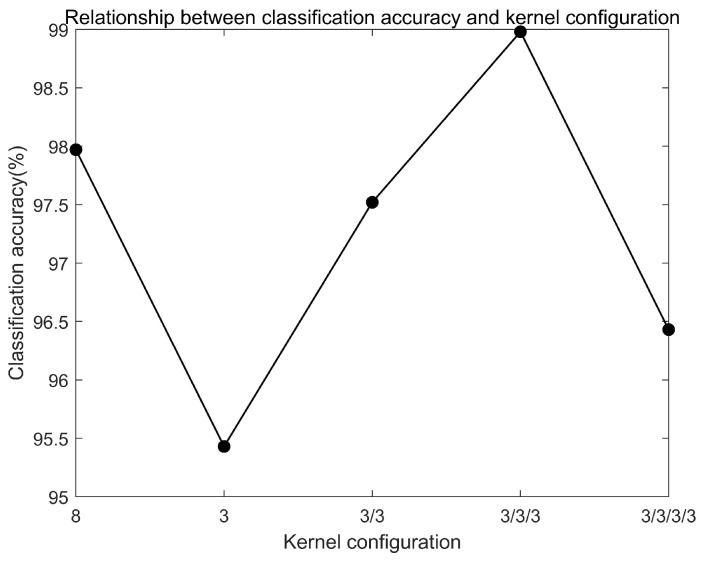
Relationship between classification accuracy and kernel configuration.

The size and number of convolution kernels determines the features extracted preliminarily in CN. Reasonable design of convolutional layer can improve the performance of CN. As mentioned in [25], several layers with small kernels are better than a single layer with large kernels. Therefore, the small kernels of size 3 are used except the first layer. The performance of different numbers of kernels are compared and shown in Figure 10. The kernel size of first layer is fixed at 64 and the latter layers are shown in Figure 10. When the latter layers are set as 3/3/3, the accuracy is the highest. Therefore, the kernels are set as 2 × 64, 1 × 3, 1 × 3, 1 × 3.

According to the above discussions, the configurations of the proposed capsule network for experiments are shown in Table 2. In the training process of the proposed model, the total loss is the combination of two outputs: the loss of classification and the loss of reconstruction. To reduce the effect of reconstruction loss, we multiply by a weight λ=0.5. Adam algorithm with default lr = 0.001 is used to train the model. Batch size is 24 and epoch number sets as 120. In addition, L2 regularization operation is applied to prevent overfitting.

### 4.4. Results and Discussions

In this part, the dataset shown in Table 1 is used to verify the proposed scheme. In addition, WDCNN (a wide first-layer deep convolutional neural network) proposed in [26] is used to compare with CN. The models are trained repeatedly with ten trials for each case.

**Figure 11:**
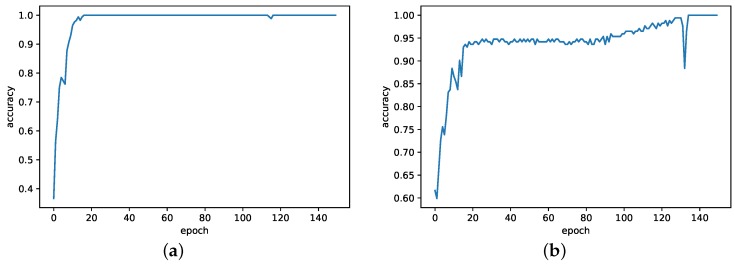
Training process of different models on A-a dataset (**a**) CN and (**b**) WDCNN.

First, to test the diagnostic performance of CN under the same rotational speed, dataset with the “no processing” (A) in Table 1 is used. It should be noted that the training and test sets are both from data with overlap for each rotational speed. The ratio of training set to test set is 4:1. The results are shown in Table 3 and Figure 11. Average and variance of the test accuracies in each case are listed in a format of “average ± variance”. As shown in Table 3, the CN model achieves accuracies of “100 ± 0.00” in all three cases, while WDCNN has misjudgments in all three cases. In particular, The proposed scheme improves by 2.27% of average when the speed is 1500rpm. In addition, comparing the training process of two methods, shown in Figure 11, the proposed scheme remains stable after 20th epoch. However, WDCNN-based method shows slightly turbulence after 30th epoch. Therefore, the CN model has a slight improvement on diagnosis accuracy and stability when the training and test data are from the same rotational speed, while the WDCNN model still is a good method for fault diagnosis under this scenario.

**Figure 12:**
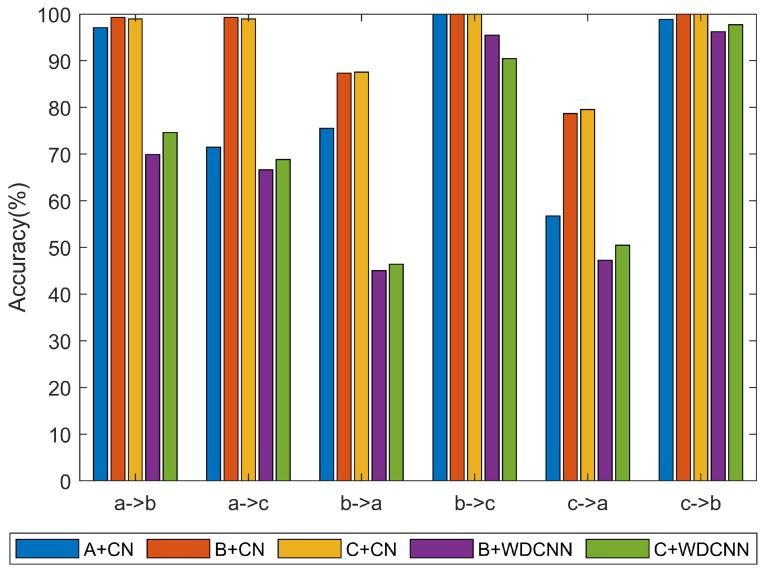
Comparison of the accuracy of different methods at different rotational speeds.

To reflect the prominent property of the proposed scheme under different rotational speeds, the vibration signals from sensors 3 and 4 are fused as input of CN. For comparison, we replace the data fusion methods and model in turn. Dataset with three fusion method are used in this part. It should be noted that the training set is from the dataset with overlap under one speed while test set is from the dataset with no overlap under another speed. The results are shown in Table 4 and Figure 12. The time is average value of each trial. There is a 300 rpm speed gap between settings b and c, while a 1000 rpm speed gap between settings a and c. Following, two discussions will be made.

(1) Comparisons on the No 1,2,3 in Table 4. They are all performed using the CN model. First, for No 2 and No 3, datasets B and C is inputted into CN, respectively. The only difference is the data fusion method using two sensors. The results show that the test accuracies of these two data fusions have small difference overall. In addition, the training time of two data fusion methods is 154 s and 152 s, and is very close. Secondly, comparing No 1 to No 3, the input data are from one sensor and two sensors, respectively. The results show that the test accuracies using two sensors have a clear enhancement. In particular, for “a→c” and “c→a” scenarios, which have a relatively large speed gap (1000rpm), the average accuracies improve by 27.50% and 22.80% respectively. As for the training time, it reduces by 10s on the contrary. These results demonstrate that the two data fusion methods based on CN model with two sensors achieve satisfactory performance for rolling bearing diagnosis under different rotational speeds.

(2) Comparing the No 2,3 to No 4,5 in Table 4. They are performed using CN and WDCNN model, respectively. For No 2 and No 4, the only difference is the classification network. The test accuracy using CN model have an improvement of 4.53% for “b→c” scenario. As the speed gap increases, namely “a→b” and “a→c”, the test accuracies improve by 29.38% and 32.68% respectively. Comparing the training time, CN spend more time due to its special structure. Considering the great accuracy improvement, spending more time is worthy. There is also a similar result between No 3 and No 5. The results show that the CN model is more suitable for fault diagnosis than CNN using two-directional sensors, especially at large speed gap.

Therefore, according to the above experiment and discussions, it demonstrates that joint use of two-direction signals together with CN is the best choice for fault classification under different rotational speeds scenario.

## 5. Conclusions

In this paper, we proposed a 2D data input capsule network scheme for fault diagnosis of rolling bearing. We aim to eliminate the influence of speed by taking advantage of capsule network with capturing the spatial relationship from the input. Experimental data from three different speeds are used to verify the performance of the proposed method. According to the results of the experiments, it may conclude that:

(1) The CN model can achieve a better performance than WDCNN when the training and test data are from the same rotational speed and only one sensor is used.
(2) When two-directional sensors are used, the proposed scheme can largely get rid of the influence of different rotational speeds for a significant improved diagnostic accuracy.
(3) The two different data fusion methods of 2D data have a little impact on the results of bearing fault diagnosis.

For future work, the performance of the proposed scheme under fluctuating speeds will be further studied.

## Figures and Tables

**Table 1:** Description of bearing datasets.

		Health State	Normal	Inner Race Fault	Rolling Element Fault	Outer Race Fault
		Label	1	2	3	4
A/B/C	set a (800 rpm)	overlap	212	212	212	212
		no overlap	32	32	32	32
	set b (1500 rpm)	overlap	212	212	212	212
		no overlap	32	32	32	32
	set c (1800 rpm)	overlap	212	212	212	212
		no overlap	32	32	32	32

**Table 2:** Details of the proposed model.

No.	Layer Name	Kernel Size/Stride/Filters	Output Shape	Padding
1	Convolution1	2 × 64/(1,8)/16	(1,256,16)	(0,28)
2	Convolution2	1 × 3/(1,1)/32	(1,125,32)	(0,1)
3	Convolution3	1 × 3/(1,1)/64	(1,125,64)	(0,1)
4	Convolution4	1 × 3/(1,1)/64	(1,125,64)	(0,1)
5	Reshape	-	(2048,8)	No
6	Squash	-	(2048,8)	No
7	Digitcaps	-	(4,16)	No
8	Length(output1)	-	(4)	No
9	Mask	-	(64)	No
10	Decoder(output2)	-	(512)	
		-	(1024)	
		-	(4098)	
11	Reshape	-	(2,2048,1)	

**Table 3:** Accuracies of different methods tested at the same speed.

Method	a ${}^{1}$	b	c
A + CN ${}^{2}$	100 ± 0.00	100 ± 0.00	100 ± 0.00
A + WDCNN	99.48 ± 0.58	97.73 ± 1.49	98.43 ± 2.42

${}^{1}$ Meaning that the model is trained and tested under the same speed; ${}^{2}$ Known as “(data processing method)+(model)”.

**Table 4:** Accuracies and time of different methods tested at different rotational speeds.

No	Method	a→b ${}^{1}$	a→c	b→a	b→c	c→a	c→b	Time
1	A + CN	94.09 ± 3.91	71.48 ± 2.39	75.53 ± 3.88	100 ± 0.00	56.73 ± 9.56	98.83 ± 0.41	162 s
2	B + CN	99.30 ± 0.58	99.30 ± 0.94	87.34 ± 2.57	100 ± 0.00	78.67 ± 2.33	100 ± 0.00	154 s
3	C + CN	98.98 ± 0.98	98.98 ± 1.04	87.58 ± 2.56	100 ± 0.00	79.53 ± 3.72	100 ± 0.00	152 s
4	B + WDCCN	69.92 ± 8.96	66.64 ± 16.56	45.00 ± 5.58	95.47 ± 2.80	47.27 ± 9.86	96.25 ± 1.46	41 s
5	C + WDCCN	74.61 ± 4.47	68.83 ± 7.53	46.41 ± 7.24	90.47 ± 9.94	50.47 ± 4.39	97.73 ± 1.93	38 s

${}^{1}$ Meaning that the model is trained and tested under the different speeds.
